# Intravenous injection of extracellular vesicles to treat chronic myocardial ischemia

**DOI:** 10.1371/journal.pone.0238879

**Published:** 2020-09-11

**Authors:** Laura A. Scrimgeour, Brittany A. Potz, Ahmad Aboul Gheit, Yuhong Liu, Guangbin Shi, Melissa Pfeiffer, Bonnie J. Colantuono, Neel R. Sodha, M. Ruhul Abid, Frank W. Sellke

**Affiliations:** Division of Cardiothoracic Surgery, Department of Surgery, Cardiovascular Research Center, Rhode Island Hospital, Brown University Warren Alpert Medical School, Providence, RI, United States of America; Emory University, UNITED STATES

## Abstract

**Background:**

Mesenchymal stem cell-derived extracellular vesicles (EVs) appear to be a very exciting treatment option for heart disease. Here, we used a swine model of chronic myocardial ischemia to evaluate the efficacy of a less-invasive method of injection of EVs via a peripheral intravenous route.

**Methods:**

Sixteen Yorkshire swine underwent placement of an ameroid constrictor on the left circumflex (LCx) artery at age 11 weeks to induce chronic myocardial ischemia. Two weeks later, they were divided into two groups: control (CON; n = 8), and intravenous injection of EVs (EVIV; n = 8). At 18 weeks of age, animals underwent final analysis and euthanasia. The chronically ischemic myocardium (LCx territory) was harvested for analysis.

**Results:**

Intravenous injection (IV) of EVs induced several pro-angiogenic markers such as MAPK, JNK but not Akt. Whereas IV injections of EVs decreased VEGFR2 expression and inhibited apoptotic signaling (caspase 3), they increased expression of VEGFR1 that is believed to be anti-angiogenic. Injection of EVs did not result in an increase in vessel density and blood flow when compared to the control group.

**Conclusions:**

Although IV injection of EVs upregulated several pro-angiogenic signaling pathways, it failed to induce changes in vascular density in the chronically ischemic myocardium. Thus, a lack of increase in vascular density at the doses tested failed to elicit a functional response in ischemic myocardium.

## Introduction

Despite numerous advances in prevention, diagnosis and treatment of cardiovascular disease, ischemic heart disease continues to increase in prevalence and remains the leading cause of mortality worldwide [1]. Furthermore, while catheter-based interventions are able to help an increasing number of patients with compromised coronary arteries, there are many patients whose disease burden is so diffuse that it is not amenable to percutaneous treatments. Additionally, even with surgical coronary artery bypass grafting, many patients retain suboptimal cardiac function or are unable to undergo grafting procedures [2, 3]. Therefore, the exploration of alternative and adjunctive therapies for ischemic heart disease is essential.

Stem cells offer a potential method for the treatment for ischemic cardiomyopathy. However, there are concerns related with stem cell therapies including immune-activation causing the need for immunosuppression, cell death, lack of honing capabilities, and the need for re-implantation of new cells to achieve a long-lasting effect on myocardial ischemia [4, 5]. To avoid immunosuppression, the need for time-intensive and invasive intra-operative harvesting for autologous stem cell transfers would be required [6]. These drawbacks make stem cell therapies a less-palatable option for much of the large population in need of cardiac revascularization. Furthermore, there is little data to suggest long-term in-vivo survival of transplanted stem cells into myocardium [7].

Recently, secretion products of stem cells known as extracellular vesicles (EVs) have been identified as carrying miRNAs, mRNAs, cytokines, growth factors and angiogenic proteins, making them a promising target for ischemic treatments [8, 9]. These vesicles have been shown to migrate to sites of injury and reprogram endogenous repair systems while limiting local inflammation. EVs are defined by their size and mechanism of release: microvesicles are released by budding off the plasma membrane and are large sized particles of >200nm; exosomes are endosomes that are released from intraluminal vesicles and are smaller in size ranging from 50-200nm [10]. Given the complicated terminology, we use the term extracellular vesicles to indicate our use of both microvesicles and exosomes. One of the exciting strengths of using extracellular vesicles is that their small size and non-cellular treatment allow flow through capillaries associated with cell-based therapies.

EVs have shown some promise of improved function in both small and large animal models of myocardial infarction [11–14]. However, none of these studies specifically address chronic myocardial ischemia, which likely has farther-reaching therapeutic benefits than only addressing myocardial infarction. Furthermore, to our knowledge, there are no studies evaluating peripheral intravenous injection of EVs. For widespread applications, minimizing the invasiveness of procedures for translation to human studies would be optimal. Here, we analyze the effect of extracellular vesicles administered via intravenous injection in a porcine model of chronic myocardial ischemia.

## Materials and methods

Sixteen intact male Yorkshire swine (Tufts University, Boston, MA) underwent placement of an ameroid constrictor (Research Instruments SW, Escondido, CA) on the left circumflex artery at age 11 weeks to induce chronic myocardial ischemia. They were then divided into two groups: control (CON; n = 8) and intravenous injection of extracellular vesicles (EVIV; n = 8). The EVIV group received intravenous injections of extracellular vesicles two weeks after the placement of the ameroid constrictor (age 13 weeks). Animals underwent euthanasia at 18 weeks of age, five weeks after treatment with intravenous extracellular vesicles or no treatment, and ischemic myocardium was harvested for Western blot analysis. Blood was also collected at the time of harvest and analyzed for cytokine levels.

### Surgical interventions

Animals received an intramuscular injection of telazol (4.4mg/kg) to induce anesthesia which was then maintained after endotracheal intubation with 0.75–3.0 MAC concentration of inhaled isoflurane. During the procedure, the animals were mechanically ventilated at 12–20 breaths per minute.

#### Myocardial perfusion

Microspheres with various isotope labeling were injected to determine myocardial blood flow. During the ameroid placement procedure, the left circumflex artery was occluded for two minutes as determined by ST elevations on the electrocardiogram monitor, during which 5 cc of gold microspheres (Biophysics Assay Laboratory, Worcester, MA) were injected into the left atrium. At the time of harvest, prior to removal of the heart, 5 cc of either lutetium, samarium, or europium-labelled microspheres were injected into the left atrium while withdrawing 10 cc of blood from the right femoral artery. Injection of samarium was done while pacing the heart at 150 beats per minute to determine blood flow during tachycardia. The heart was then divided into tissue sections based on anatomy in relation to the left anterior descending artery (non-ischemic tissue) and left circumflex artery (ischemic tissue) and small amounts of each section were weighed, dried and analyzed for content of microspheres.

#### Metabolic parameters

Prior to each surgical intervention, blood glucose was measured, and each animal received a glucose tolerance test involving intravenous injection of 50% dextrose (0.5g/kg). Blood glucose levels were determined at 30 and 60 minutes following the dextrose injection. At the time of each procedure, weight, length and circumference was determined for each animal. Blood samples were taken either from the left atrium or femoral artery catheter to analyze cholesterol and liver function parameters.

#### Ameroid constrictor placement

After antibiotic prophylaxis, animals underwent a left mini-thoracotomy through which the left atrium was identified and retracted, exposing the left circumflex artery. Here, an ameroid constrictor was placed (1.75–2.5mm in diameter, depending on the size of the artery) and the incision was closed in a layered fashion. All animals received pain control and a five-day course of antibiotics and aspirin to prevent infection or thromboembolic events respectively.

#### Intravenous injection of extracellular vesicles

Animals were sedated and an intravenous catheter was placed into the auricular vein or saphenous vein. Two milliliters of extracellular vesicles were injected slowly into the IV catheter, and then flushed with saline. Atipamezol (0.5–1.0mg/kg) was then given as a sedation reversal agent.

#### Cardiac harvest

Five weeks following injection of extracellular vesicles, animals were placed under general anesthesia and a median sternotomy was performed to expose the heart. Hemodynamic parameters were gathered via a pressure-volume catheter (Millar Inc., Houston, TX) and after euthanasia by exsanguination; myocardial tissue was collected for further analysis. The tissue was sectioned into numbered pieces based on location from the left anterior descending artery during the tissue sectioning. Section 3 is located at the area perfused by the left circumflex and therefore the most ischemic after ameroid constriction of the left circumflex artery. Therefore Section 3 myocardial tissue was used for all of the “ischemic” tissue referred to in this manuscript.

All experiments were approved by the Institutional Animal Care and Use Committee of Rhode Island Hospital and all animals were cared for in compliance with “Principles of Laboratory Animal Care” formulated by the National Society for Medical Research and the “Guide for the Care and Use of Laboratory Animals” (NIH publication no. 5377–3 1996).

#### Isolation of extracellular vesicles

**Fig 1:**
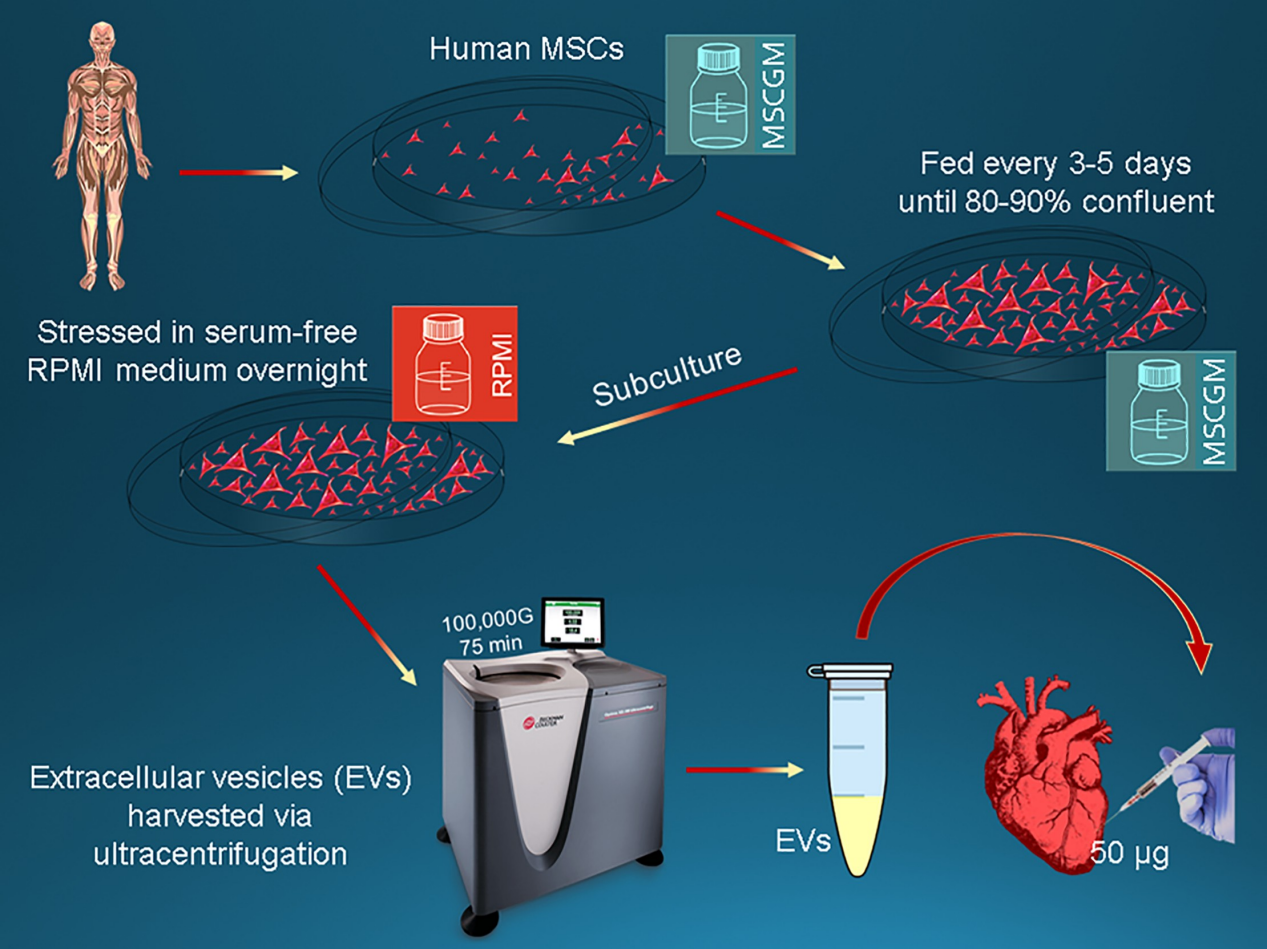
Extracellular vesicle isolation. Schematic of cell culturing of human bone-marrow derived mesenchymal stem cells, stress, and isolation of extracellular vesicles.

Human bone marrow-derived mesenchymal stem cells (Lonza, Allendale, NJ) were cultured in MSCGM Bulletkit media (Lonza) and grown to confluence after several passages for expansion. The day prior to isolation, the media was removed and replaced with serum-free RPMI media for overnight incubation. Extracellular vesicles were then harvested via ultracentrifugation (ThermoScientific Sorvall WX Ultra Series Centrifuge with a Sorvall Surespin rotor, ThermoScientific, Waltham, MA) at 100,000g for 75 minutes, then resuspended and spun at 100,000g in PBS for 30 minutes, then resuspended in 10% DMSO [Fig 1]. Protein was quantified using a Micro BCA Protein Assay Kit (ThermoFisher Scientific, Waltham, MA). Each animal received 2 mL containing 50 μg of protein containing the extracellular vesicles in sterile 0.9% saline.

#### Electron microscopy

After ultracentrifugation of the EVs, the pellet was resuspended in 2% paraformaldehyde. 5μL resuspended pellets were then placed on Formvar-carbon coated EM grids and adsorbed for 20 minutes, then washed in PBS. The grids were then transferred to a 50μl drop of 1% glutaraldehyde for 5 minutes, then washed eight times in distilled water. The samples were then contrasted in uranyl-oxalate solution for 5 minutes, then embedded in methyl cellulose for 10 minutes on ice. The grids were removed, air-dried, and observed under the electron microscope (Philips 410 Transmission Electron Microscope, Amsterdam, Netherlands) at 80 kV.

#### Western blot analysis

Homogenized tissue lysates were fractionated on 4–20% Tris-Glycine gels (Novex™ Midi Protein Gels, Invitrogen, Carlsbad, CA) then transferred to polyvinylidene difluoride membranes (Millipore, Bedford, MA). Protein concentration was determined using a radio-immunoprecipitation assay (Pierce BCA Protein Assay Kit, ThermoFisher Scientific, Waltham, MA). Primary antibodies [phosphoinositide 3-kinase (PI3K), Akt, phosphorylated Akt, extracellular signal-regulated kinase (ERK1/2), microtubule associated protein kinase (MAPK), B cell lymphoma-2 (BCL-2), phosphorylated BCL-2, Bcl-2-associated death promoter (BAD), phosphorylated BAD, caspase 3, caspase 9, cleaved caspase 9, c-Jun N-terminal kinase (JNK), vascular endothelial growth factor receptor 1 (VEGF-R1), and vascular endothelial growth factor receptor 2 (VEGF-R2), all from Cell Signaling, Danvers, MA] at a 1:1000 dilution were incubated overnight at 4°C. Membranes were washed, and then a secondary antibody at appropriate dilution was added and incubated for one hour at room temperature. GAPDH was added to all membranes for loading control. Chemiluminescent images were viewed and recorded using a digital camera (GBox, Syngene, Cambridge, England) and analyzed using Image-J software (National Institutes of Health, Bethesda, MD) to quantify band densitometry. Data are reported as arbitrary light units representative of protein band density normalized to GAPDH and fold-change values compared to controls.

#### Immunofluorescence

At the time of harvest, tissue from ischemic myocardium was fixed in 10% formalin and 24-hours later, transferred to 70% ethanol. Slides were stained with cluster of differentiation 31 (CD31, Abcam, Cambridge, UK) and α-smooth muscle actin (α-SMA, Cell Signaling, Danvers, MA), as previously described [16]. After paraffinization, slides were deparaffinized, rehydrated, then blocked with peroxide, rinsed, and blocked with an antibody at 1:50 dilution overnight. A second antibody was incubated for an hour at room temperature after rinsing. Images were captured at 20x objective with a Nikon Eclipse TE2000-U microscope (Nikon Instruments, Melville, NY). These images were then analyzed using Image-J software (National Institutes of Health, Bethesda, MD).

### Statistical analyses

GraphPad Prism 5.0 Software (GraphPad Software Inc., San Diego, CA) was used to perform a Mann-Whitney two-tailed t-test. Data are presented as mean ± SEM. A p value of <0.05 was used for statistical significance.

## Results

### Metabolic parameters

Metabolic parameters including glucose levels, bilirubin, protein, albumin, CRP, insulin levels, cholesterol levels (HDL, LDL, triglycerides) and fructosamine were measured via arterial blood sampling and no significant difference was seen between the groups at the time of harvest. The alkaline phosphatase was higher in the CON than the EVIV group (p = 0.04).

### Myocardial perfusion

**Fig 2:**
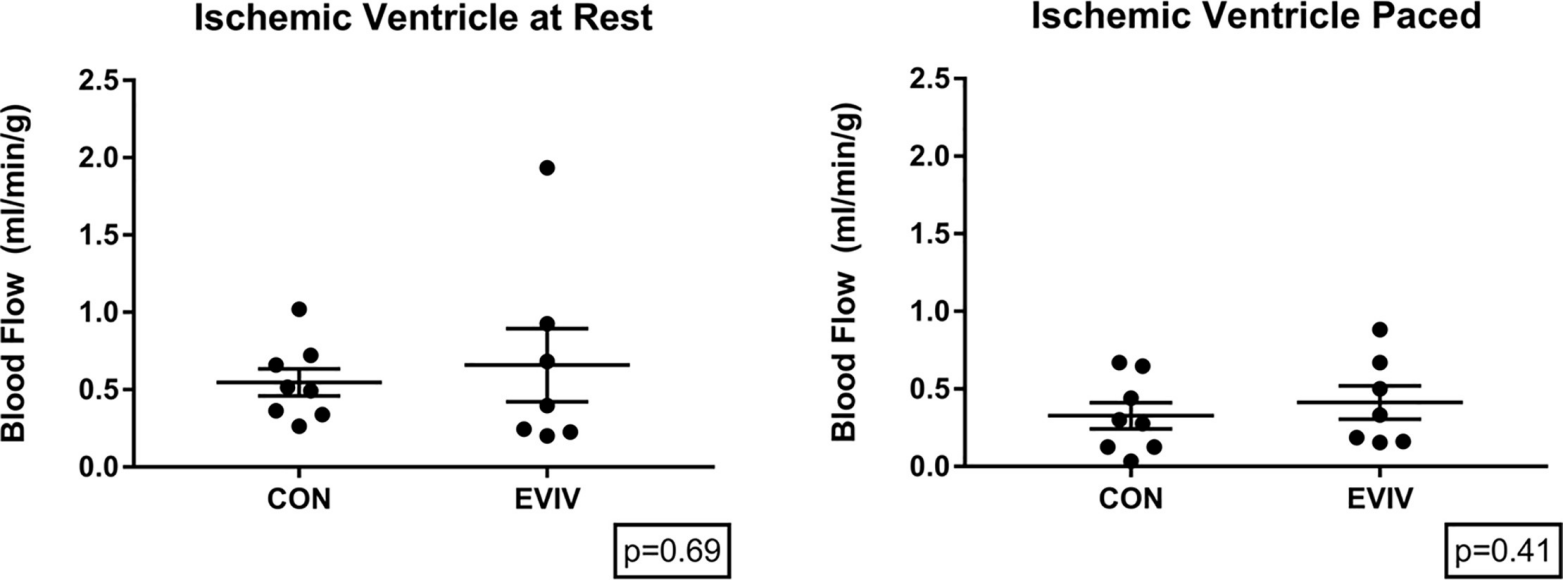
Intravenous injection of extracellular vesicles. No significant change was seen in blood flow (ml/min/g) either at rest or during pacing. CON: Control group, EVIV: Extracellular Vesicle Intravenous injection group.

Blood flow to the ischemic area of the myocardium (perfused by the chronically occluded left circumflex) was not significantly different for those treated with intravenous injection of extracellular vesicles (p>0.9). When blood flow was assessed during cardiac pacing to 150 beats per minute, there was again no significant difference observed from the control group. One pig from each group was excluded because the result was an outlier at more than 2 standard deviations from the mean. [Fig 2]

### Apoptotic protein signaling

**Fig 3:**
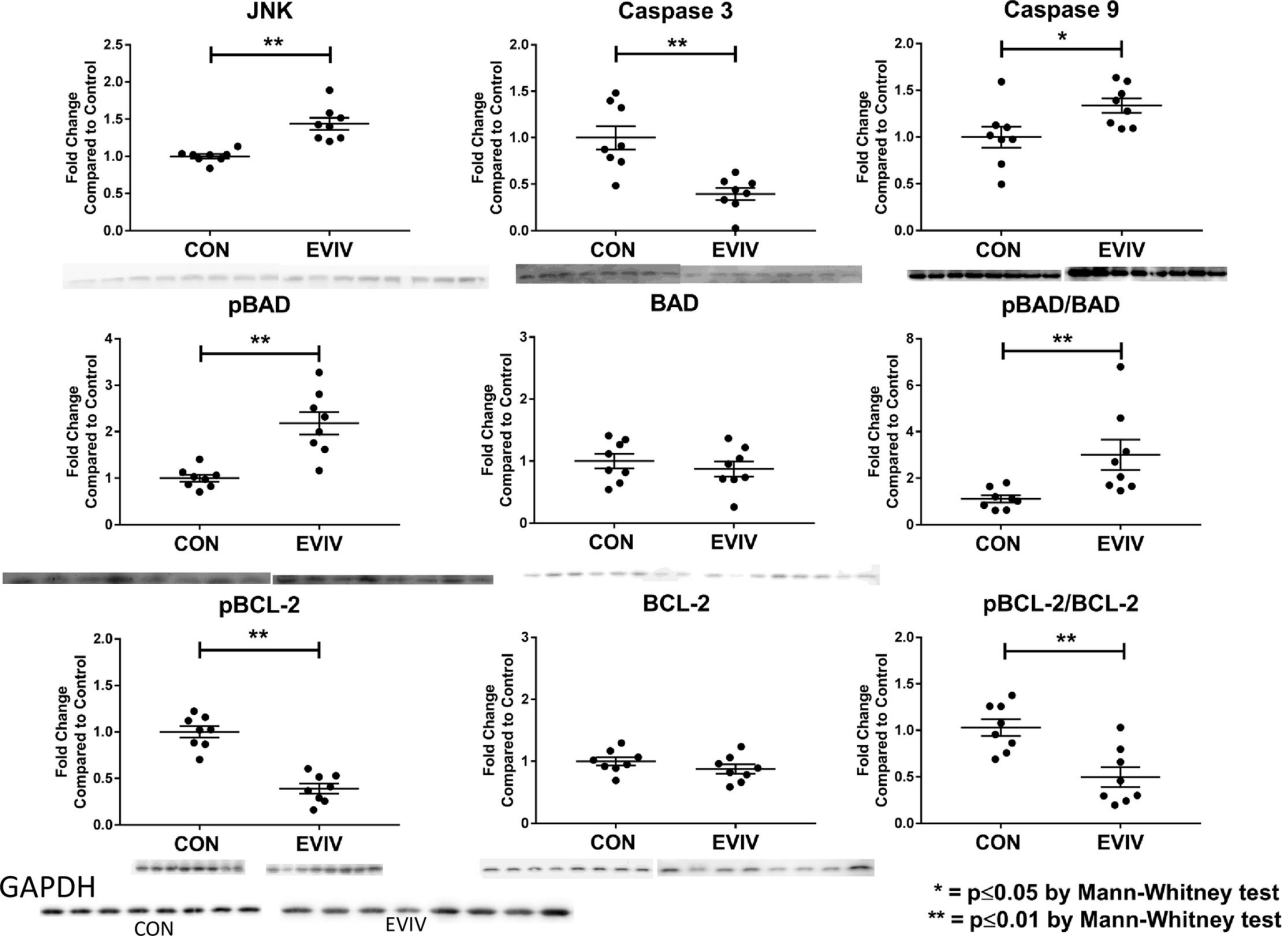
Decrease in apoptosis in the ischemic myocardium. There is an increase seen in JNK, pBAD, and pBAD/BAD with a decrease in pBCL-2, pBCL-2/BCL-2, and Caspase 3. Overall, this suggests a decrease in apoptosis and increase in cell-survival. CON: Control group, EVIV: Extracellular Vesicle Intravenous injection group, *p≤0.05 and **p<0.01 via Mann-Whitney test.

There were significantly upregulated levels of phosphorylated BAD compared to the control (p = 0.002), despite no significant change in the overall amount of Akt (p>0.9). JNK was also significantly upregulated (p = 0.008). While we do not see a significant change in BCL-2, phosphorylated BCL-2 was significantly decreased compared to the control (p = 0.004). Furthermore, there is a decrease in activation of the caspase cascade via decreased caspase 3 (p = 0.001). Interestingly, there is an increase in caspase 9 (p = 0.02). The overall significant decrease in caspase 3, however, suggests downregulation of apoptosis. [Fig 3 and Table 1]

**Table 1:** Markers of apoptosis and angiogenesis.

	CON	EVIV	p-value
JNK	1	1.43 ± 0.08	0.0002*
Caspase 3	1	0.395 ± 0.065	0.0011*
Caspase 9	1	1.34 ± 0.07	0.0207*
Cleaved Caspase 9	1	0.91 ± 0.17	0.5054
Cleaved Caspase 9/Caspase 9	1.08 ± 0.16	0.71 ± 0.15	0.1049
pBAD	1	2.18 ± 0.24	0.0003*
BAD	1	0.87 ± 0.12	0.5737
pBAD/BAD	1.11 ± 0.15	3.01 ± 0.65	0.0019*
pBCL-2	1	0.39 ± 0.05	0.0002*
BCL-2	1	0.88 ± 0.07	0.2345
pBCL-2/BCL-2	1.03 ± 0.09	0.50 ± 0.11	0.0047*
MAPK p42	1	1.95 ± 0.18	0.0011*
VEGF-R1	1	3.35 ± 0.56	0.0006*
VEGF-R2	1	0.53 ± 0.08	0.0019*
MAPK	1	0.90 ± 0.04	0.5737
pMAPK	1	1.8 ± 0.12	0.0148*
TGF-β	1	1.25 ± 0.09	0.0148*
eNOS	1	0.087 ± 0.056	0.2786
pAkt	1	0.85 ± 0.09	0.1049
Akt	1	0.95 ± 0.10	0.5054
pAkt/Akt	1.03 ± 0.07	0.90 ± 0.09	0.2786

All markers are load controlled to GAPDH and normalized to CON and listed as mean ± SEM. P values determined via at two-tailed t-test (Mann-Whitney). CON: Control group, EVIV: Extracellular Vesicle Intravenous injection group.

*p≤0.05 and

**p<0.01 via Mann-Whitney test.

### Angiogenic protein signaling

**Fig 4:**
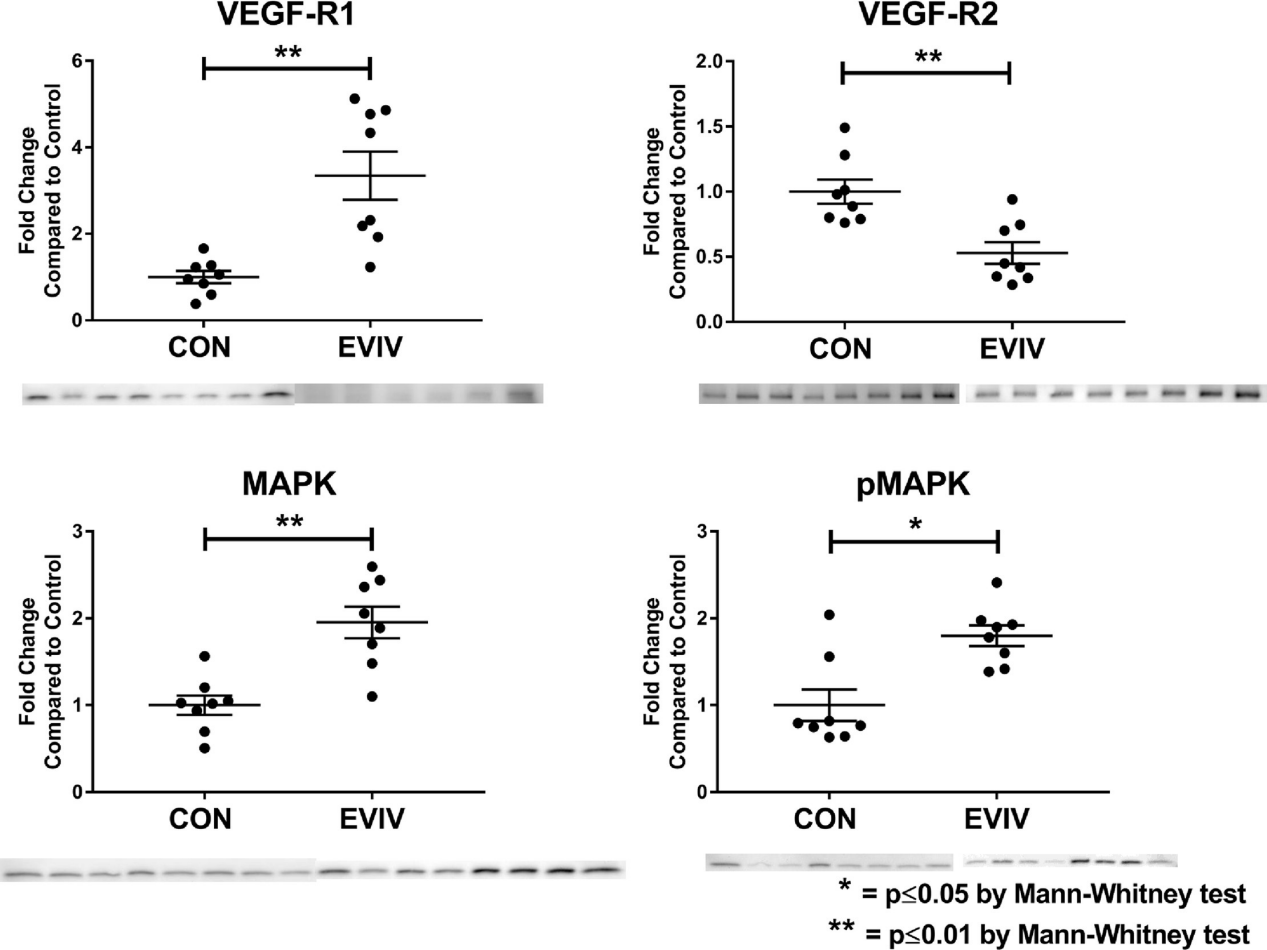
Angiogenesis signaling in the ischemic myocardium. The EVIV group had a significant increase in MAPK, pMAPK, and VEGF-R1. CON: Control group, EVIV: Extracellular Vesicle Intravenous injection group, *p≤0.05 and **p<0.01 via Mann-Whitney test.

VEGF-R1 was significantly increased over the control group (p = 0.001), however VEGF-R2 was significantly downregulated (p = 0.002). Additionally, we observed a significant upregulation of in MAP kinase (p = 0.001) and an increase in phosphorylated MAP kinase (p = 0.01). [Fig 4 and Table 1].

### Size differences between stressed and non-stressed EVs

**Fig 5:**
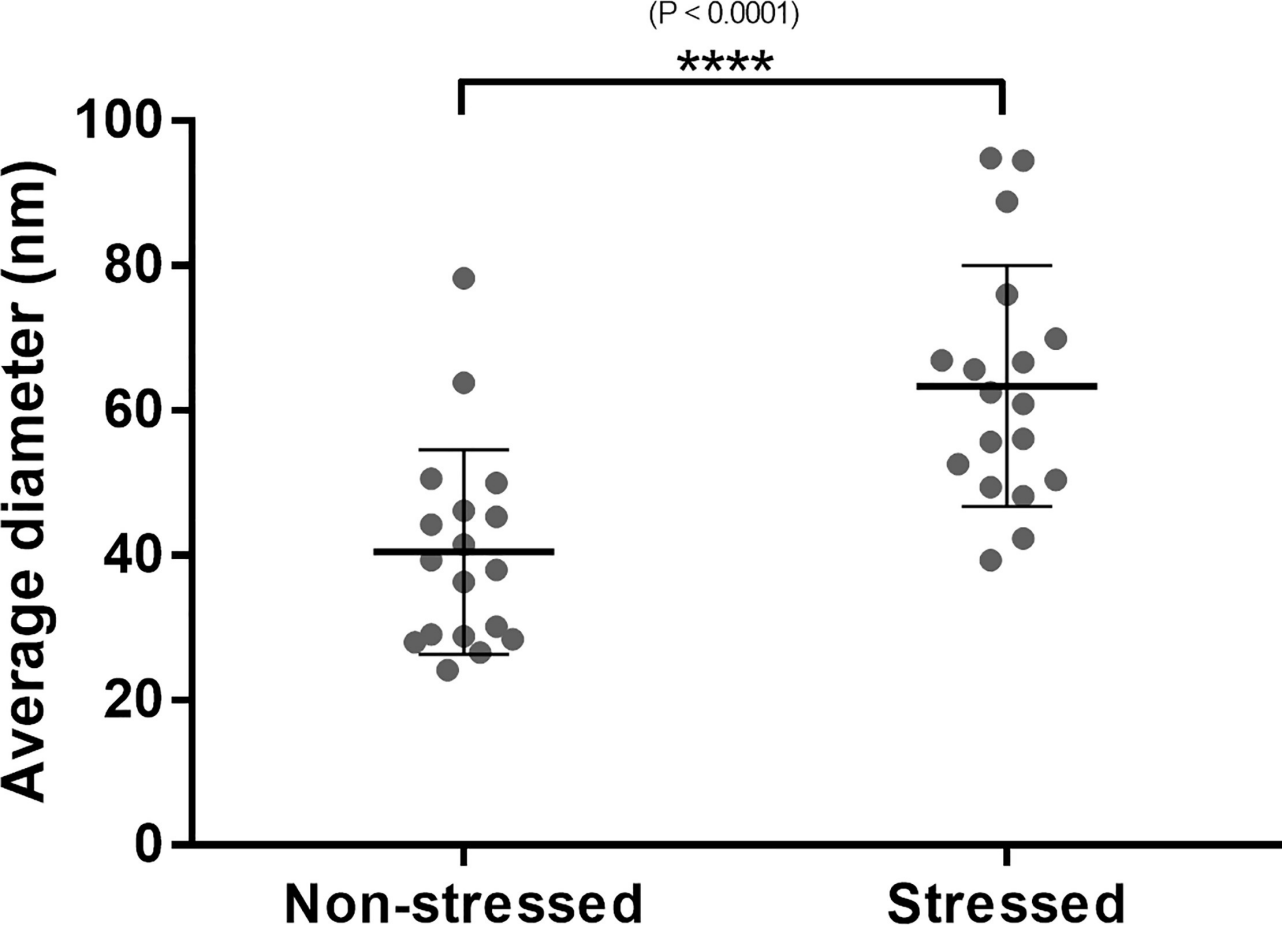
Stressed conditions cause a size increase in EVs.

**Fig 6:**
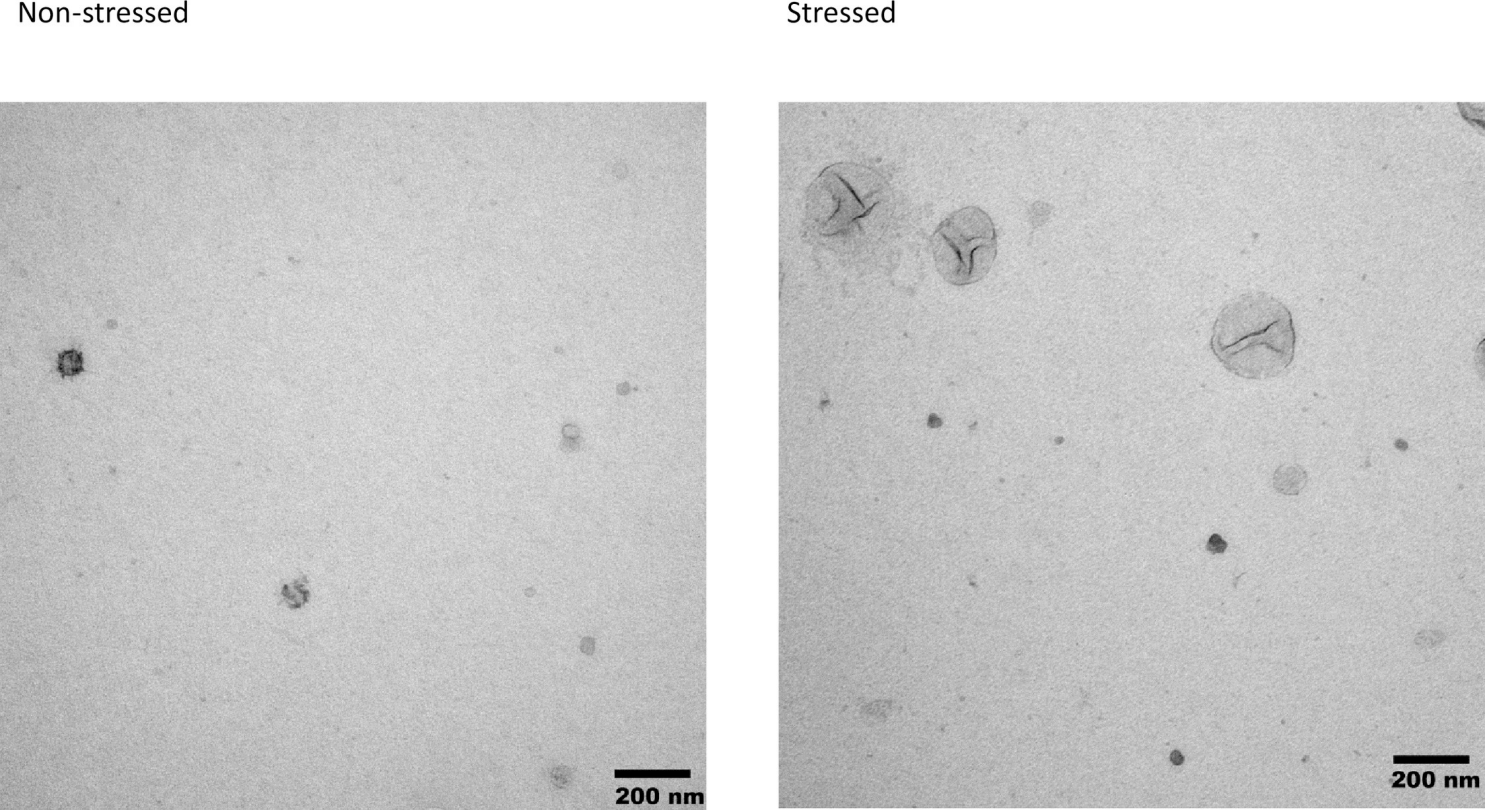
Electron microscopy images of extracellular vesicles under stressed and non-stressed conditions.

Mesenchymal stem cells which are subjected to stressed conditions for 24 hours produce significantly larger EVs than cells which are not subjected to this stress. [Figs 5 and 6]

### Myocardial arteriolar density

**Fig 7:**
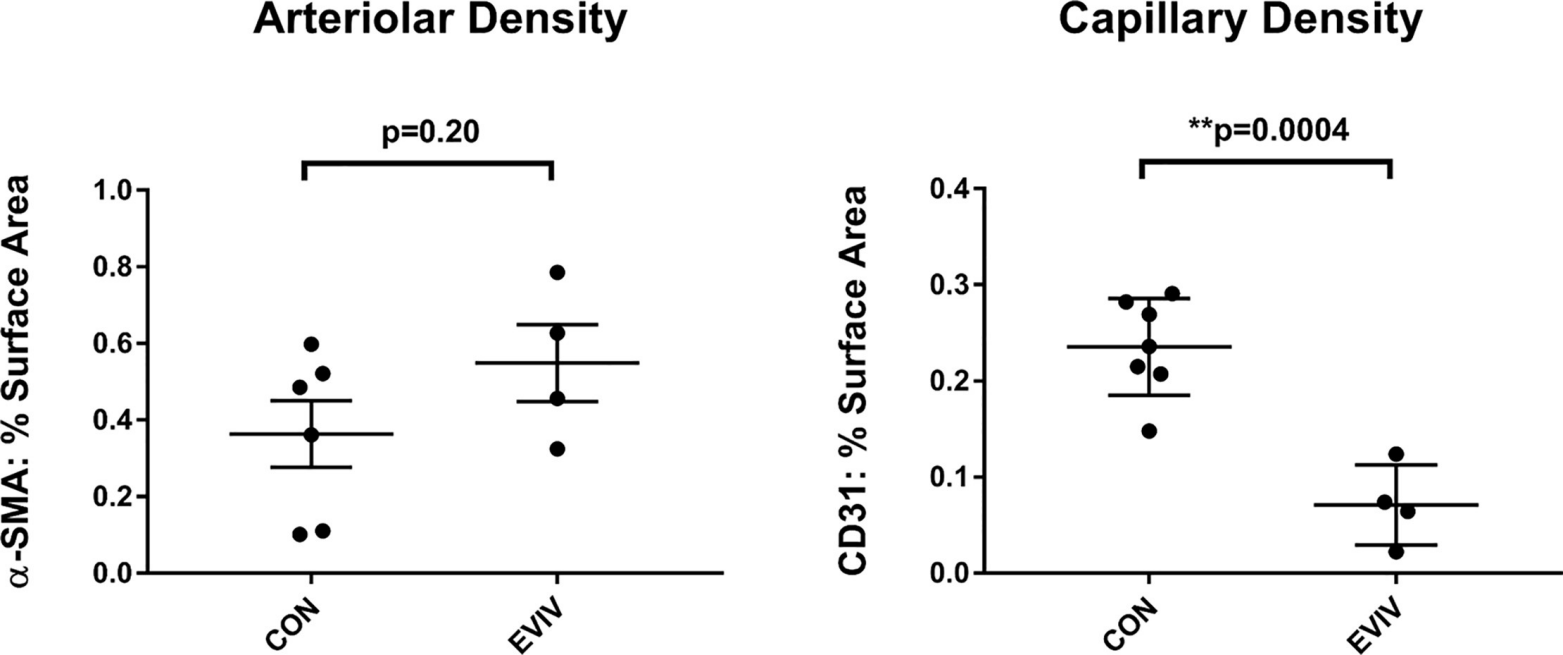
Vascular density as a measure of angiogenesis. There was no difference in arteriolar density between groups and rather a decrease in capillary density. CON: Control group, EVIV: Extracellular Vesicle Intravenous injection group, **p<0.01 via Unpaired t-test.

There was not an increase in capillary density counts as determined by CD31 staining in the animals treated with EVs via IV when compared to the controls, rather, there was actually a significant decrease in CD31 staining (EVIV: 0.07±0.02 vs CON: 0.24±0.02, p = 0.004). [Fig 7] Furthermore, there was no significant difference in arteriolar density between the two groups as determined by α-SMA staining, although there was a trend toward increase arteriolar density. This suggests a relative lack of angiogenesis in the ischemic myocardium (EVIV: 0.55±0.1 vs CON: 0.36±0.09, p = 0.2).

## Discussion

Coronary artery disease is becoming ever more prevalent in the U.S. Chronic myocardial ischemia often exists silently in patients for years prior to the presentation of an acute myocardial infarction. Therefore, our model of using an ameroid constrictor on the left circumflex artery to induce chronic myocardial ischemia over a several week period has translational advantages when compared with an acute ligation animal model of myocardial ischemia. The goal of our research is to identify a therapy that will treat chronic ischemia with the goal of prevention of an acute event [14, 15].

This study sought to accomplish both the evaluation of a less-invasive delivery model for treatment of chronic myocardial ischemia while also further elucidating mechanisms of action through which EVs are able to increase perfusion to ischemic myocardium in the setting of chronic myocardial ischemia. This is the first study to our knowledge to evaluate a less-invasive means of delivery of EVs as a means of treatment of chronic myocardial ischemia. Our group has previously demonstrated improved vascular density and myocardial perfusion with the intramyocardial injection of extracellular vesicles [16]; however, the invasive nature of an additional thoracotomy limits the translational applications of this research to future human studies. Other groups have evaluated intracoronary infusion via catheterization, however not in a model of chronic ischemia [15, 17]. Therefore, we sought to explore a less-invasive, intravenous infusion approach of delivering treatment. Unfortunately, we did not find a significant increase in the blood flow to the ischemic area while using a less-invasive, intravenous approach. One plausible explanation for this would be that we used similar dosages of EVs for IV injection as has been used in previous studies of intramyocardial injections; since IV provides a less direct application of therapy, we plan on repeating the experiment using an increased dose of EVs in the IV group. Other explanations include an inactivation of EV’s when injected into the blood, versus the myocardial as was done in other experiments where a clear benefit was evident [16, 18]. It is interesting that EV’s actually decreased the density of capillaries in the ischemic myocardium and there was a tread toward an increase in arteriolar density. This suggests that the intravenous injection may have a differential effect on angiogenic signaling compared to that observed when the EV’s are injected directly into the myocardium.

Improved blood flow to areas of ischemia remains an overarching goal in the treatment of myocardial ischemia. Treatments which improve angiogenesis are an obvious target for achieving this goal. VEGF-R2 is a pro-angiogenic modulator while the actions of VEGF-R1 are more dynamic. We observed a decrease in VEGF-R2 in the EVIV group, which may explain why we do not see increased blood flow in that group compared to the control group. Furthermore, we did not observe an increase in capillary nor arteriolar density in tissue sections of ischemic myocardium in the EVIV treated group, which further demonstrates the lack of angiogenesis in this tissue. VEGF-R1 has been shown to affect vascular development differentially at different stages. It is initially inhibitory to sprouting, but later becomes essential for branching and connections with other vessels [19]. Interestingly, we see an increase in VEGF-R1 but perhaps the levels are so high that they cause an overall inhibitory effect on sprouting and the initiation of the angiogenic response.

Activation of VEGF-R2 has been shown to have downstream effects on the MAPK/ERK1/2 pathways and inhibition of this interaction is associated with inhibition of angiogenesis [20]. Upregulation of intracellular signaling cascades including MAP kinase are essential in carrying out intracellular functions required for angiogenesis. It is very interesting to note the higher levels of MAPK and pMAPK in the animals treated with IV injections of extracellular vesicles The lack of change observed in blood flow in the IV-injected group in this study could suggest the need for a direct myocardial treatment as an initial treatment, but the opportunity for repeated administrations of extracellular vesicles through peripheral intravenous administration at future time points to augment the beneficial effects. Collectively, this suggests that while increased myocardial blood flow was not observed in the EV-injected group at the time of harvest, this group had a greater intracellular angiogenic response via the MAP kinase pathway and expression of VEGF receptors that may be indicative of a delay in response to the peripheral circulation of extracellular vesicles in comparison to local injection at the area of chronic ischemia.

Akt is known to inhibit the apoptosis via mitochondrial and calcium-induced cell death pathways by inhibition of BAD and the caspase cascade [21, 22]. Similarly, the MAPK/ERK pathway is involved in regulating intracellular signaling involved in apoptosis [23]. Here, we note an increase in JNK, pBAD, and pBCL-2, which collectively work to downregulate apoptosis and promote cell survival. The significant downregulation of caspase 3 further supports evidence demonstrating a decrease in apoptosis in the ischemic tissue after treatment. Taken together, the findings suggest that extracellular vesicles promote cell survival and intrinsic repair mechanisms in injured cardiac muscle tissue.

### Limitations

One of the major limitations to this study is that we only used one dose of extracellular vesicle injection at one time point. Further studies will need to be done to determine a dose-response curve, as well as the potential need for repeated treatments and optimal timing. Additionally, while porcine cardiac physiology closely resembles human cardiac physiology, extrapolation of data may be limited by inter-species variability. Furthermore, we kept the animals in each group to a minimal number and therefore also only used male, intact animals and there may be sex differences that must be taken into account.

## Conclusion

This study demonstrates that while intravenous injection of EVs does not directly increase blood flow, they may serve to decrease inflammation and apoptosis in ischemic myocardium, which could be of benefit over time.

## Supporting information

S1 File (PPTX)
