# Expression of Concern: Intravenous injection of extracellular vesicles to treat chronic myocardial ischemia

**DOI:** 10.1371/journal.pone.0357070

**Published:** 2026-08-27

**Authors:** The PLOS One Editors

After this article [1] was published, concerns were raised regarding Figs 3, 4, and the methods. Specifically:

- The BCL-2 EVIV panel of Fig 3 and the VEGF-R1 Con panel of Fig 4 appear similar to each other.
- The animal methodology does not report the humane endpoints used, details on animal monitoring, or the IACUC approval code for these experiments.
- The underlying images provided in S1 File in [1] do not appear to match the corresponding panels presented in Figs 3 and 4.

The corresponding author stated that the BCL-2 EVIV panel in Fig 3 is incorrect. They provided a corrected Fig 3 (S2 File) and noted that the underlying image for the correct BCL-2 EVIV panel in Fig 3 is provided in the S1 File published with [1].

With regards to the animal experiments, the first author stated that the IACUC approval code is no longer available, and that animals were monitored twice daily post-operatively for three days, then 2–3 times per week until the harvest procedure. The first author further stated that the following signs were used as humane endpoint criteria for euthanasia: clinical condition that does not respond to treatment (e.g., infected surgical site); delayed wound healing; dehiscence of surgical site; difficulty in ambulation which renders the animal unable to access food/water; persistent and progressive dermatitis or self-trauma; CNS signs such as tremors, seizures, circles that were not anticipated by the study plan; anorexia > 48 hours; other lesions interfering with eating or drinking; sudden behavioral change (e.g., aggression, guarding, hiding); weight loss of 20% or more from baseline at the start of the experiment or as compared to age/gender/strain matched controls; markedly discolored urine; excessive urine, or no urine; severe or refractory diarrhea or decreased fecal output > 48 hours; dehydration unresponsive to oral or parental therapy; rough hair coat, hunched posture, distended abdomen, reluctance to move, or lethargy; respiratory signs such as labored breathing, wheezing, or copious nasal discharge; and hemorrhage (blood loss) from any site that is estimated to be > 10% total circulating blood volume.

Contrary to the declaration in the Data Availability statement, the original raw data files supporting the article’s results were not provided with the article, and the first author stated these data are no longer available. As the original raw data files supporting the article’s results are no longer available, this article [1] does not comply with the PLOS Data Availability policy.

In light of the above concerns regarding the underlying images provided in S1 File in [1], and because the article does not comply with the PLOS Data Availability policy, the *PLOS One* Editors issue this Expression of Concern.

## Supporting information

S2 File The corrected Fig 3. (TIF)
