# Supplementary material for: Intravenous injection of extracellular vesicles to treat chronic myocardial ischemia
Source: PLoS One. 2020 Sep 11;15(9):e0238879. doi: 10.1371/journal.pone.0238879 (PMC7485873; doi:10.1371/journal.pone.0238879)
Supplement: S1 File — (PPTX) [file pone.0238879.s001.pptx]

## Slide 1
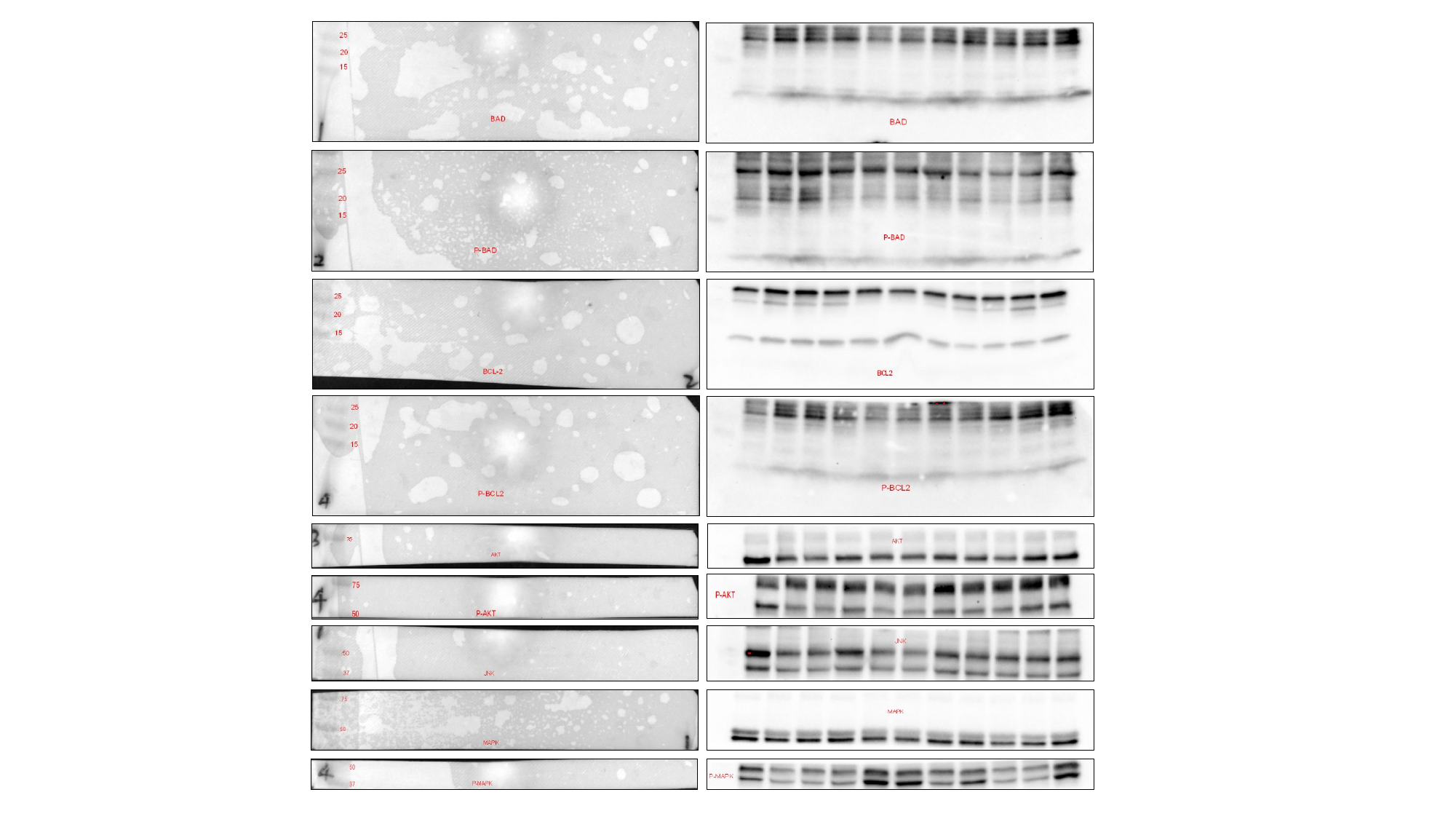

## Slide 2
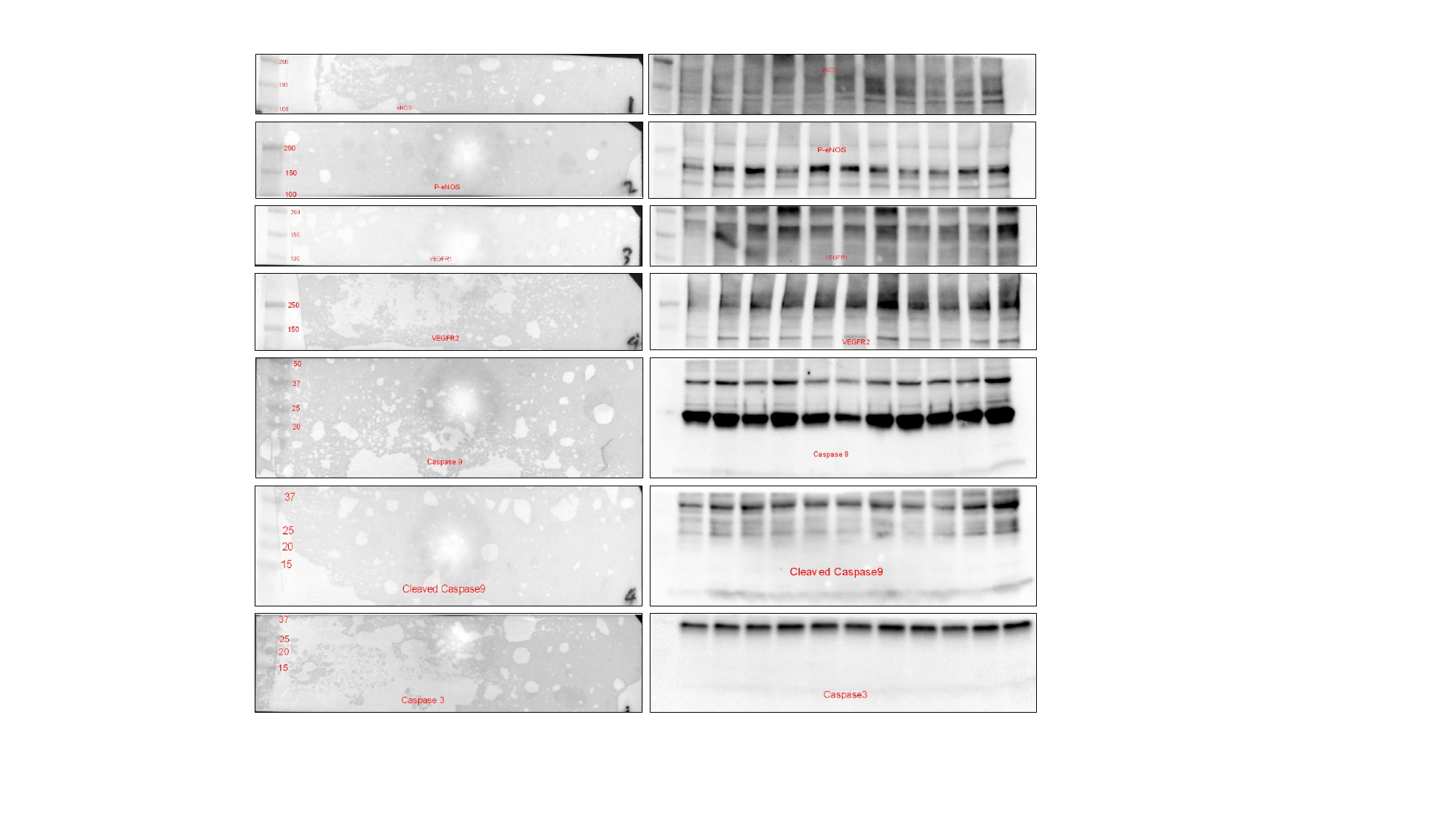

## Slide 3
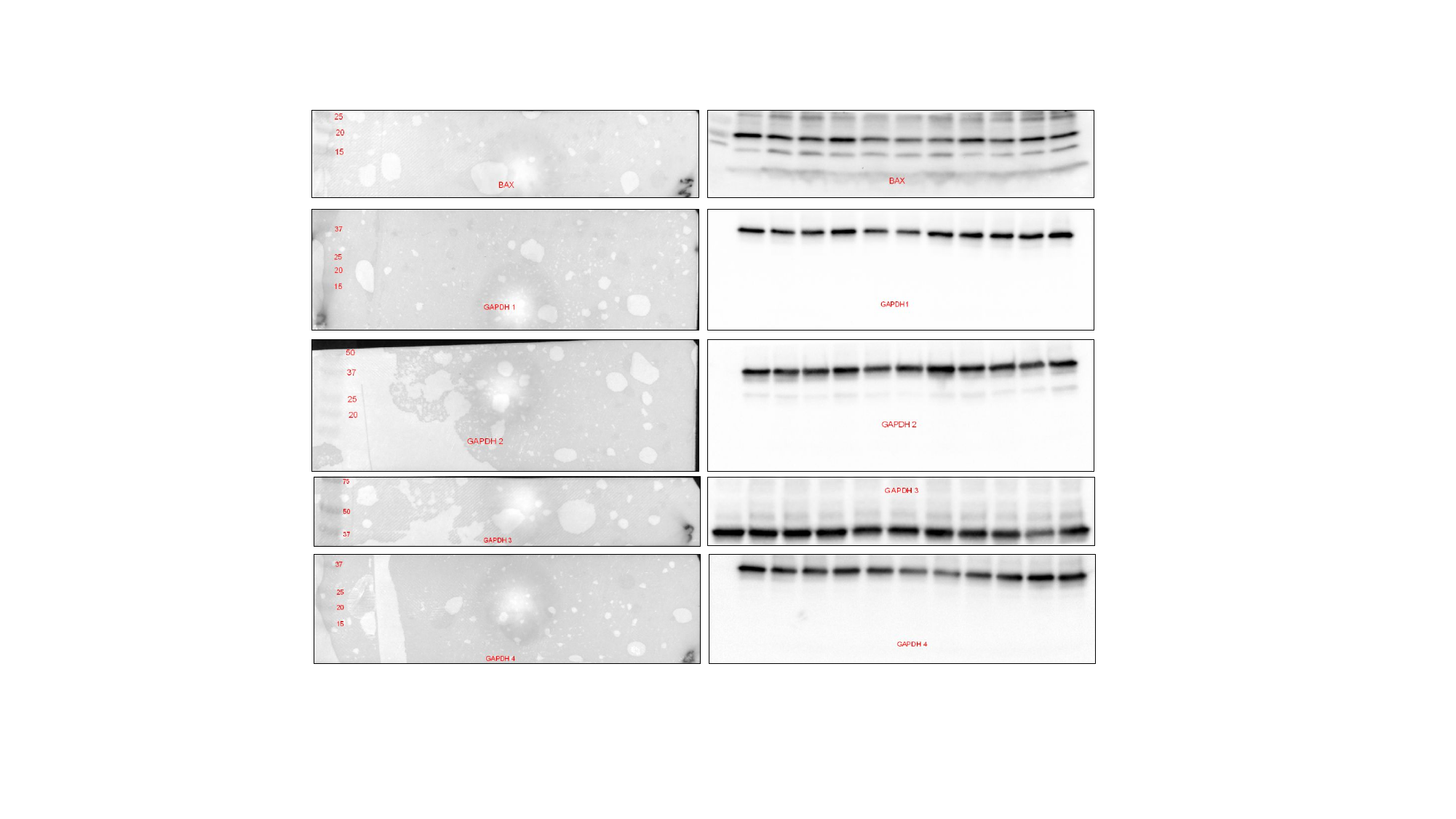

## Slide 4
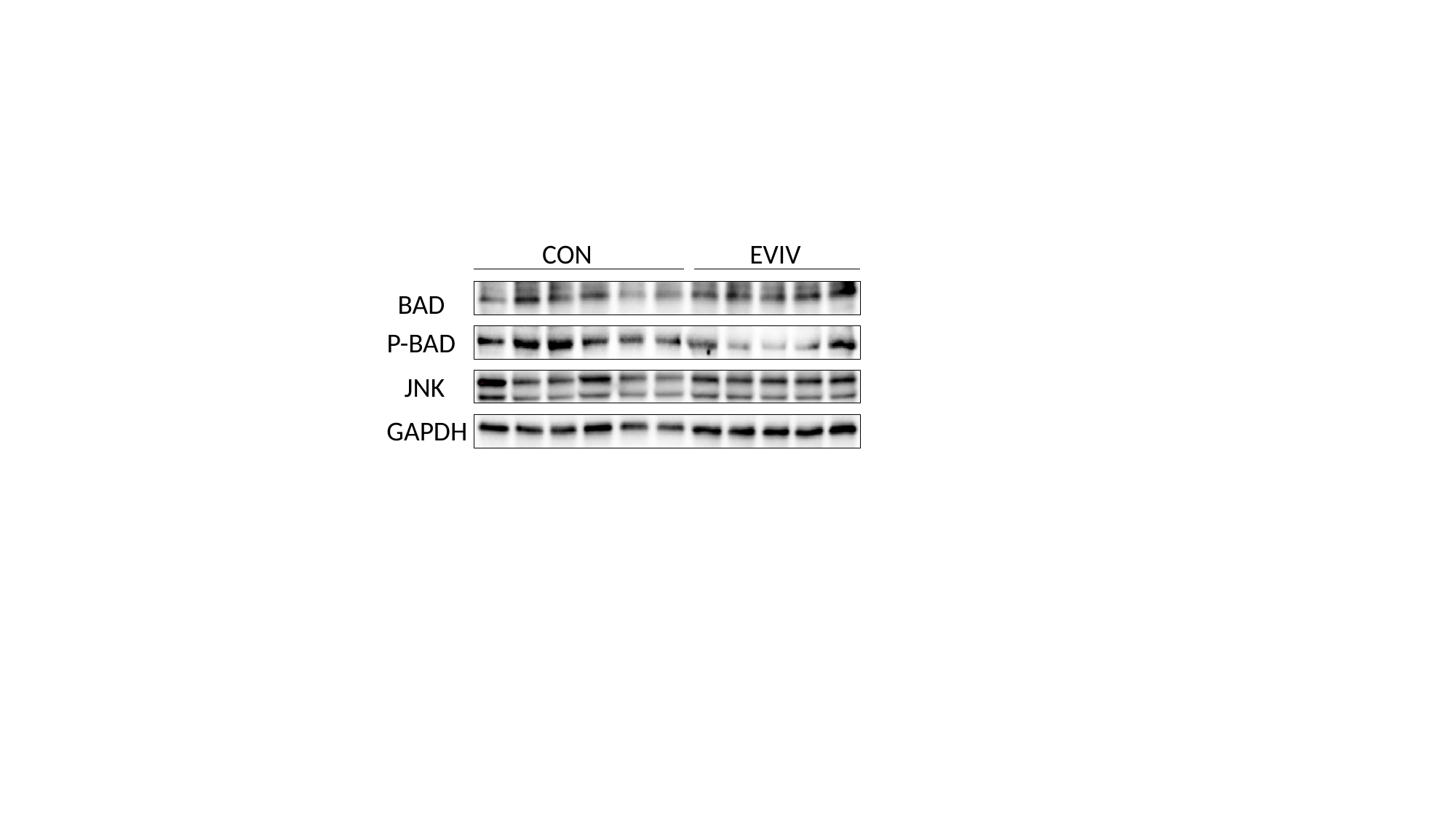

EVIV
CON
BAD
P-BAD
JNK
GAPDH

## Slide 5
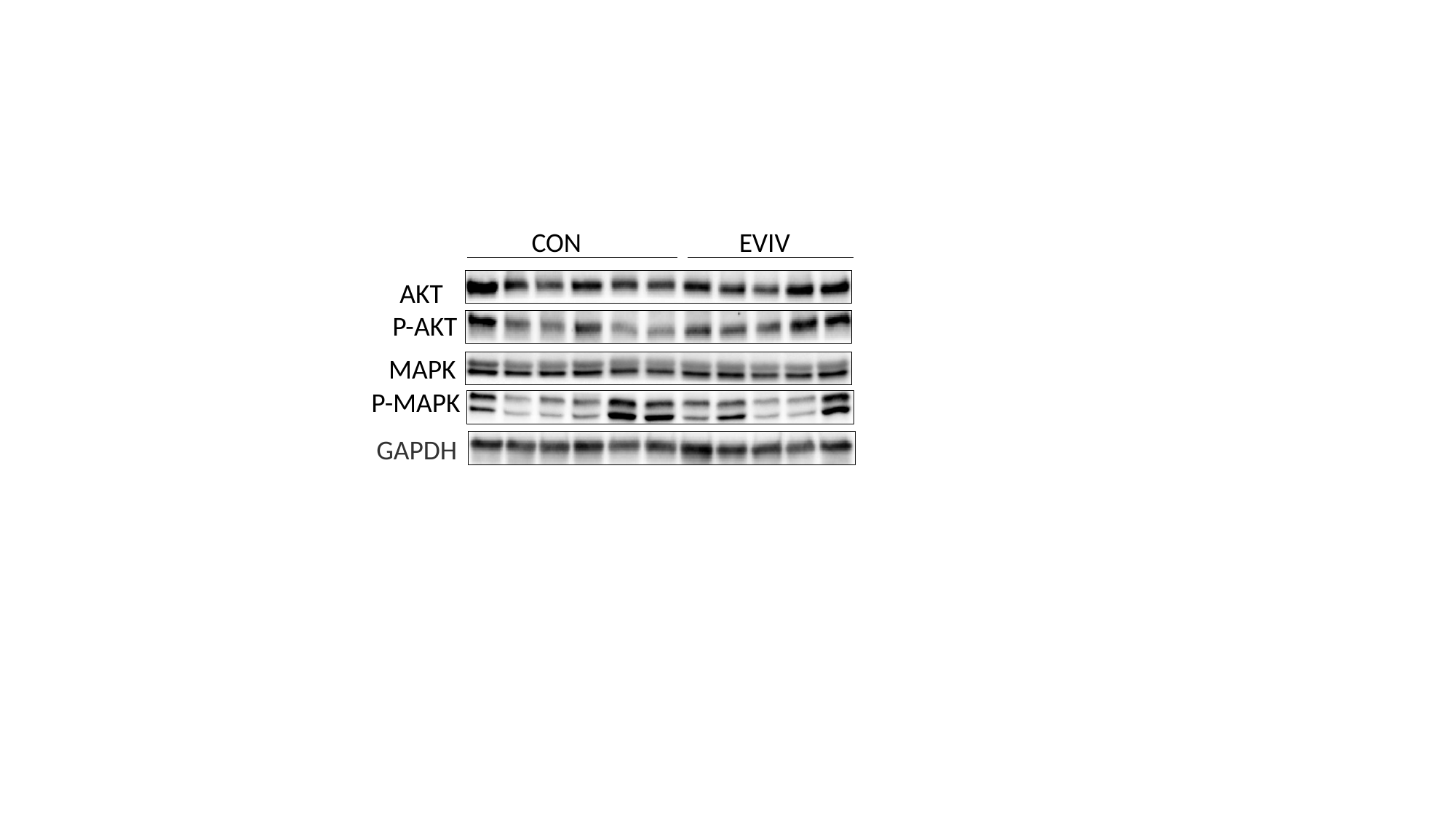

EVIV
CON
AKT
P-AKT
MAPK
P-MAPK
GAPDH

## Slide 6
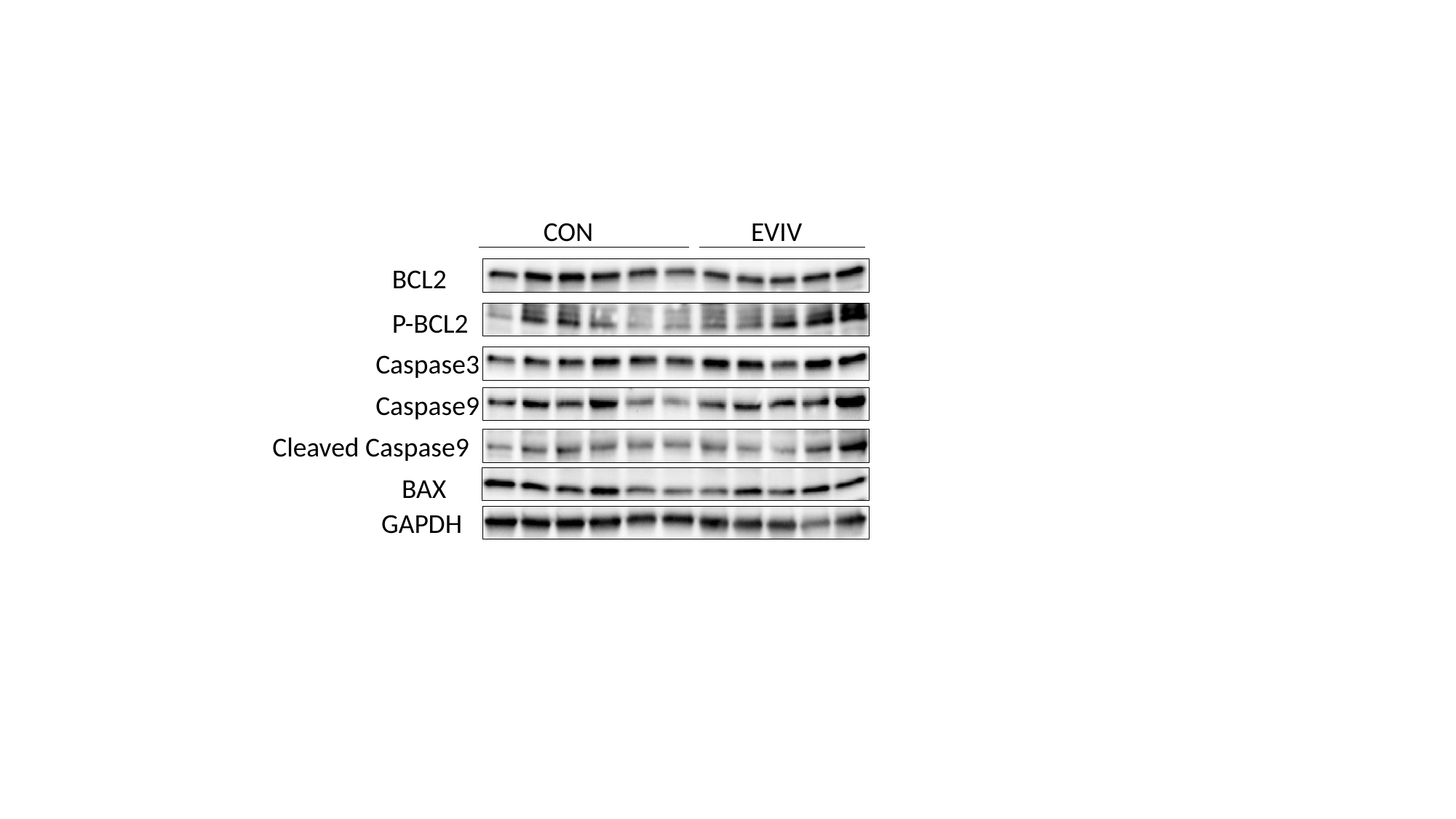

EVIV
CON
BCL2
P-BCL2
Caspase3
Caspase9
Cleaved Caspase9
BAX
GAPDH

## Slide 7
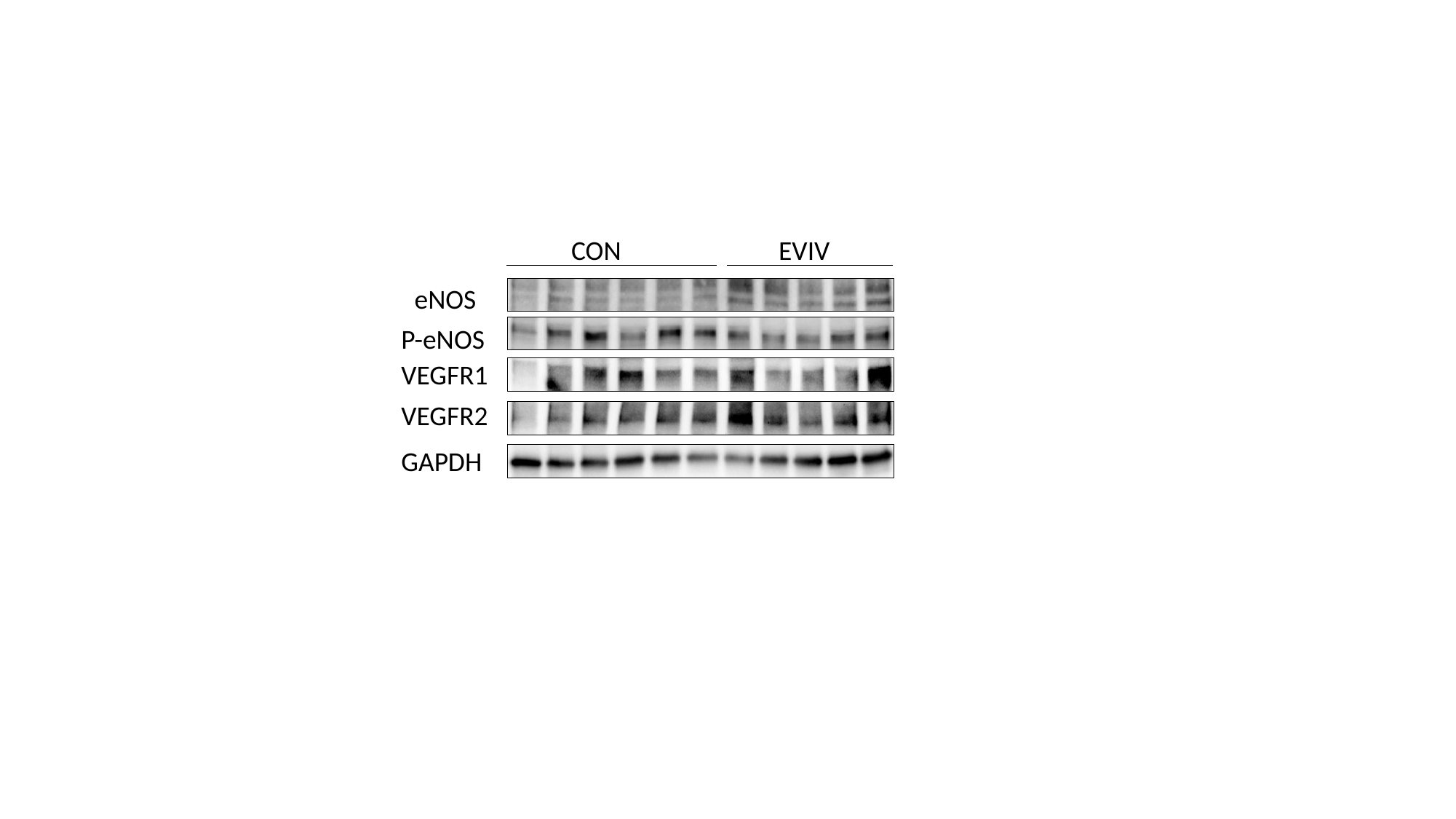

EVIV
CON
eNOS
P-eNOS
VEGFR1
VEGFR2
GAPDH
